# The role of RNA m6A demethylase ALKBH5 in the mechanisms of fibrosis

**DOI:** 10.3389/fcell.2024.1447135

**Published:** 2024-08-16

**Authors:** Ziwei Liao, Jing Wang, Mengrou Xu, Xiaoyan Li, Hongming Xu

**Affiliations:** Department of Otorhinolaryngology Head and Neck Surgery, Shanghai Children’s Hospital, Shanghai Jiao Tong University School of Medicine, Shanghai, China

**Keywords:** ALKBH5, fibrosis, m6A modification, fibroblasts, RNA modification

## Abstract

ALKBH5 is one of the demethylases involved in the regulation of RNA m6A modification. In addition to its role in the dynamic regulation of RNA m6A modification, ALKBH5 has been found to play important roles in various tissues fibrosis processes in recent years. However, the mechanisms and effects of ALKBH5 in fibrosis have been reported inconsistently. Multiple cell types, including parenchymal cells, immune cells (neutrophils and T cells), macrophages, endothelial cells, and fibroblasts, play roles in various stages of fibrosis. Therefore, this review analyzes the mechanisms by which ALKBH5 regulates these cells, its impact on their functions, and the outcomes of fibrosis. Furthermore, this review summarizes the role of ALKBH5 in fibrotic diseases such as pulmonary fibrosis, liver fibrosis, cardiac fibrosis, and renal fibrosis, and discusses various ALKBH5 inhibitors that have been discovered to date, exploring the potential of ALKBH5 as a clinical target for fibrosis.

## 1 Introduction

ALKBH5 is one of the demethylases involved in the regulation of RNA N6-methyladenosine (m6A) modification. Its main function is to remove the m6A modification from RNA (Aik et al., 2014). m6A modification is a common form of RNA chemical modification that plays important roles in RNA stability, translational regulation, and post-transcriptional modification. ALKBH5 catalyzes the reaction that converts m6A modification back to adenosine, thereby participating in the dynamic regulation of RNA (Toh et al., 2020; Yu et al., 2021). In recent years, research on ALKBH5 has attracted widespread attention and has made significant progress in the field of life sciences.

Studies on ALKBH5 have demonstrated its crucial roles in multiple biological processes. Firstly, ALKBH5 is involved in the regulation of RNA metabolic homeostasis. By regulating the level of m6A modification, ALKBH5 can influence RNA degradation rate and translation efficiency, thereby controlling gene expression level and cellular functions (Zheng et al., 2013; Yu et al., 2021). Secondly, ALKBH5 plays an important role in germ cells, participating in germ cell development and maturation processes. Studies have shown that the loss of ALKBH5 can lead to germ cell apoptosis and infertility (Tang et al., 2018; Cai et al., 2022). Furthermore, ALKBH5 is closely associated with the occurrence and progression of tumors, and its expression level in tumor cells are related to prognosis and treatment outcomes (Hu et al., 2022; Zhai et al., 2023).

**FIGURE 1:**
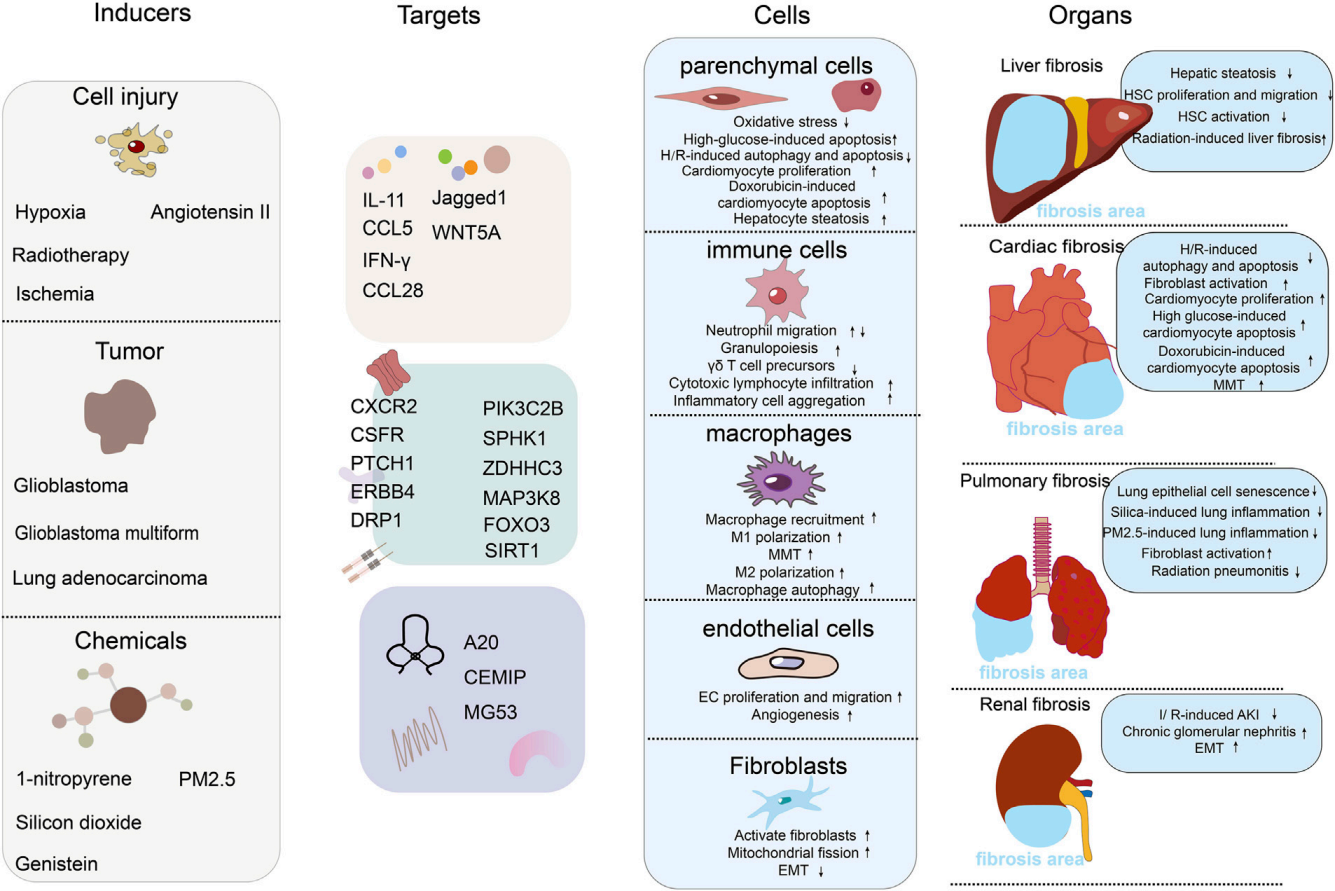
The role of RNA m6A demethylase ALKBH5 in the mechanisms of fibrosis. The figure includes 4 columns: inducers, targets, cells, organs. Inducers: this column lists the factors or conditions that can affect or activate the function of ALKBH5, including chemicals, tumor, and cell injure. Each inducer may modulate ALKBH5 activity or expression level in different ways. Targets: this column shows the targets of ALKBH5. We have divided the targets into three major categories: secreted proteins (such as interleukins, chemokines, and ligand proteins), cell membrane receptor proteins and their downstream signaling pathway proteins, and extracellular matrix proteins. Cells: this column shows the effects of ALKBH5 on different types of cells, including parenchymal cells (hepatocytes and cardiomyocytes), immune cells (neutrophils, T cells, etc.), macrophages, endothelial cells, and fibroblasts. We list the effects of ALKBH5 on cells functions. Organs: in this column, we show the effects of ALKBH5 on the fibrosis of different organs, including liver fibrosis, cardiac fibrosis, pulmonary fibrosis, and renal fibrosis. We list specific induction models and the mechanisms of fibrosis development.

In recent years, ALKBH5 has been found to play important regulatory roles in various tissues fibrosis processes, although the mechanisms of ALKBH5 in fibrosis have been reported inconsistently (Ning et al., 2020; Sun et al., 2022a; Yang et al., 2022). Therefore, this review summarizes the research progress of ALKBH5 in various fibrosis-related cells and diseases such as pulmonary fibrosis, liver fibrosis, cardiac fibrosis, and renal fibrosis. Understanding the role of ALKBH5 in fibrosis not only contributes to a deeper understanding of its biological processes but also provides potential targets for the treatment and drug development of fibrotic diseases (Figure 1).

## 2 The structure and function of ALKBH5

**FIGURE 2:**
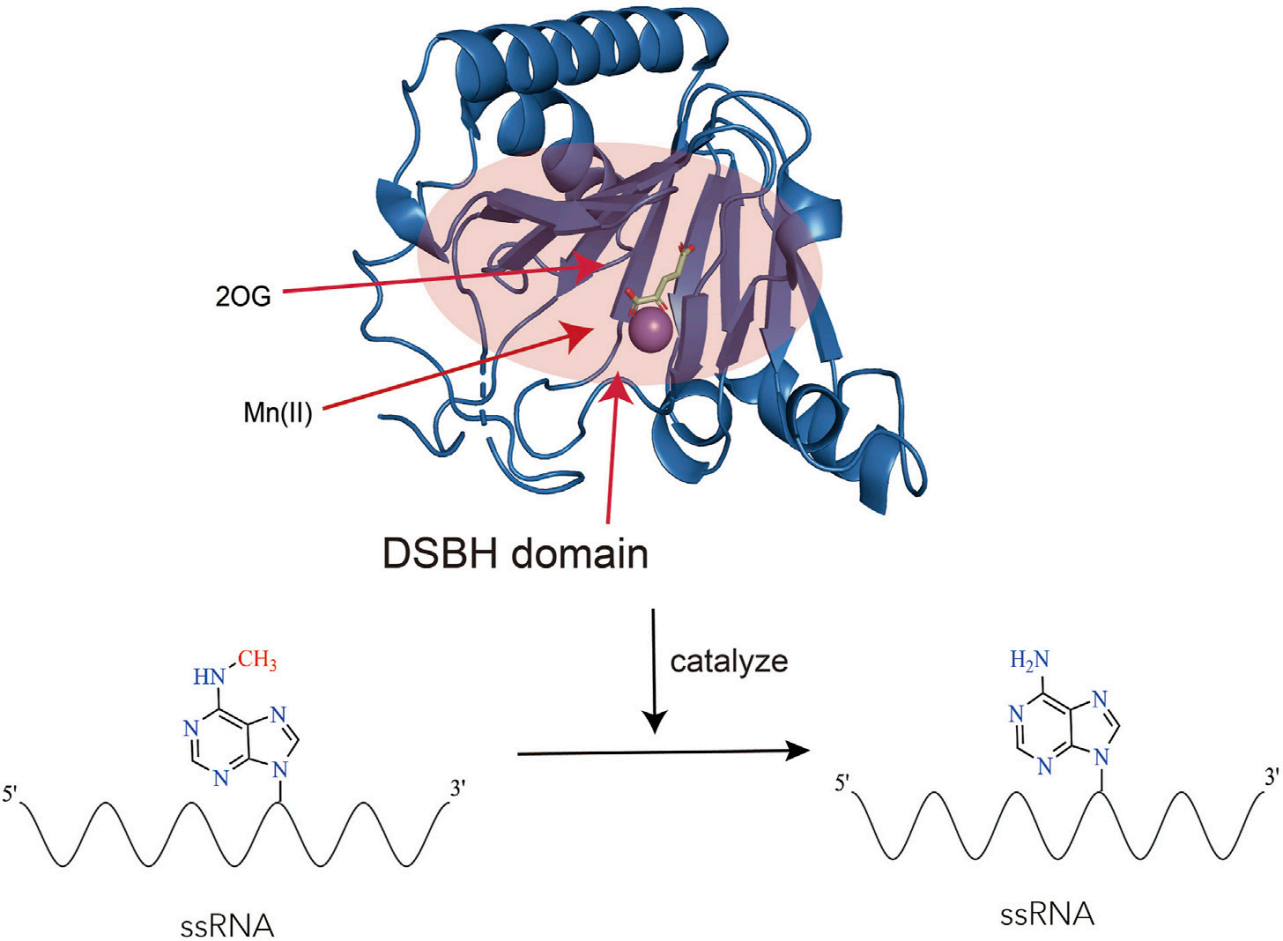
The structure and function of ALKBH5. The figure showcases the structure of ALKBH5 (Protein Data Bank ID: 4OCT) in complex with Mn(II) and 2-oxoglutarate (2OG). Mn(II) is a substitute for Fe(II) (Xu et al., 2014). The highlighted red area corresponds to the DSBH (Double-Stranded Beta-Helix) domain, which possesses the ability to bind to Fe(II), 2-oxoglutarate (2OG), and single-stranded RNA (ssRNA). Within this region, ALKBH5 performs a vital function by catalyzing the removal of N6-methyladenosine (m6A) modifications from RNA.

ALKBH5 belonged to the iron (II)- and 2-oxoglutarate-dependent AlkB oxygenase family (Aik et al., 2014). Endogenous ALKBH5 was primarily found in nuclear speckles, where facilitated mRNA processing. ALKBH5 was an mRNA-binding protein, and newly synthesized RNA was its main substrate (Meyer et al., 2012; Zheng et al., 2013; Feng et al., 2014). Structurally, the catalytic center of ALKBH5 contained a double-stranded β-helix domain (DSBH structure), consisting of 11 β strands and 5 α helices. The DSBH domain could bind to Fe^2+^ and 2OG. The binding of Fe^2+^ and 2OG directly affected the function of ALKBH5. The binding of Fe^2+^ and 2OG occurred prior to the binding of primary substrates (m6A-modified single-stranded nucleic acids) to the active site of ALKBH5. Studies had found that ALKBH5 was more disordered in solution than observed in its crystal structure. These differences were likely due to the absence of the Cys230-Cys267 disulfide bond in solution, which restricted the binding of 2OG to the “catalytic pocket” of ALKBH5. After 2OG binding to the “catalytic pocket,” the enzyme underwent a conformational change, enlarging the active site and increasing the enzyme’s affinity for substrates, allowing small molecule substrates to enter the active site (Purslow et al., 2018). Studies had also revealed the influence of Fe^2+^ on the function of ALKBH5: the octahedral structure formed by the binding of Fe^2+^ to the enzyme could promote its stability, and make residues near the key motif HX (D/E) in the catalytic pocket more hydrophobic (Feng et al., 2014). The hydrophobic environment was favorable for the enzyme to bind to substrates and catalyze reactions. Additionally, the binding of ALKBH5 to primary and secondary substrates required the mediation of Fe^2+^ (Zheng et al., 2014). Research had found that ALKBH5 could regulate ferroptosis (Lv et al., 2023). However, whether Fe metabolism and ferroptosis affect the function of ALKBH5 remained unreported at present (Figure 2).

## 3 The impact of ALKBH5 on cellular functions in fibrosis

Fibrosis referred to the excessive activation of fibroblasts, leading to the deposition of extracellular matrix components, including collagen, in response to various stimulus. The activation and transformation of fibroblasts into myofibroblasts were key events in fibrosis. Myofibroblasts could also undergo transformation from other cell types, such as epithelial/endothelial cells through epithelial/endothelial-mesenchymal transition (EMT/EndMT) and from macrophages through macrophage-to-myofibroblast transition (MMT). Additionally, various cell types, including parenchymal cells, immune cells, macrophages, and endothelial cells (ECs), played important roles in various stages of fibrosis process. Current research had found that ALKBH5 regulated the functions of these cells in different ways (Table 1).

**TABLE 1:** The impact of ALKBH5 on cellular functions in fibrosis.

Cell types		Expression^a^	Target RNAs	mRNA stability	Target pathways	Functions	Ref.
parenchymal cells	Cardiomyocytes	Up	MG53	Increased		Suppress apoptosis and cells death	Li et al. (2024a)
			SIRT1			Inhibit oxidative stress and apoptosis	Liu and Liu (2023)
			FOXO3		CDR1/Hippo	Aggravate high-glucose-induced apoptosis	Shao et al. (2022)
			ARID2			Aggravate doxorubicin-induced apoptosis	Chen et al. (2024)
	hepatocytes	Down	AXL	Decreased		Induced hepatocytes autophagy	Meng et al. (2023)
Immune cells	Neutrophils	Up	G-CSFR	Increased		Promote neutrophil migration	Liu et al. (2024)
			CXCR2, NLRP12	Increased	CXCR2, NLRP12	Promote granulopoiesis and neutrophil mobilization	Liu et al. (2022)
			PTGER4, TNC, and WNK1	Decreased^b^		Promote neutrophil migration	
	CD4^+^ T cells	Up	IFN-γ	Increased		Promote the secretion of IFN-γ	Zhou et al. (2021)
			CXCL2			Promote CD4^+^ T cell responses and neutrophil recruitment	
	γδ T cells	Up	Jagged1, Notch2	Increased		Inhibit the development of γδ T cell precursors	Ding et al. (2022)
	Treg cells	Down	CCL28	Increased		Promote Treg cell migration	Chen et al. (2023a)
Macrophages	GBM-Mø	Up	lncRNA NEAT1	Increased		Promote CXCL8/IL8 and Mø recruitment	Dong et al. (2021)
	HCC- Mø	Up	MAP3K8	Increased	JNK/ERK	Promote Mø recruitment	You et al. (2022)
	Retinal microglia	Down	A20	Decreased^b^		Enhance M1 polarization of retinal microglia	Chen et al. (2022)
	HSCs-Mø	Up	CCL5	Increased		Promote M2 polarization	Chen et al. (2023b)
	Mø	Up	IL-11	Increased		Promote MMT	Zhuang et al. (2024)
			Slamf7	Decreased		Induced Mø autophagy	Yin et al. (2024)
Endothelial cells	ECs	Up	SPHK1	Increased		Promote EC angiogenesis	Kumari et al. (2021)
		Down	WNT5A	Increased		Contribute to angiogenic phenotype	Zhao et al. (2021)
	LC-ECs	Up	lncRNA PVT1	Increased		Promote angiogenesis in LC	Shen et al. (2022)
	HCC-ECs	Up	circ-CCT3	Increased		Affect angiogenesis in HCC	Liu et al. (2023)
Fibroblasts	Fibroblasts	Up	ErbB4	Increased		Activate myofibroblasts	Yang et al. (2023)
			FOXM1	Increased	miR-320a-3p/FOXM1 axis	promoted lung fibroblast activation	Sun et al. (2022b)
	HSCs	Up	PTCH1	Increased	Hedgehog	Inhibited HSC activation	Yang et al. (2022)
			Drp1	Decreased^b^		Promote mitochondrial fission and HSC proliferation and migration	Wang et al. (2023b)

Mø, macrophages; GBM, glioblastoma multiforme; HCC, hepatocellular carcinoma; MMT, macrophage-to-myofibroblast transition; HSCs, hepatic stellate cells; ECs, endothelial cells; LC, lung cancer; NSCLC, Non-small cell lung cancer; EMT, Epithelial-Mesenchymal Transition.

^a^ ALKBH5 expression.

^b^ The difference in RNA stability was due to the different types of m6A reader.

### 3.1 The impact of ALKBH5 on parenchymal cells

ALKBH5 played significant roles in the repair process of cardiomyocytes injury, which was essentially a tissue damage repair mechanism. In general, it was believed that in adult mammals, when cardiomyocytes suffered significant and sustained damage, fibrosis was the main mechanism for repair, due to their lack of regenerative capacity (Henderson et al., 2020).

Mitsugumin 53 (MG53), also known as TRIM72, was a potential cardiac protective protein (Zhao et al., 2024). When cardiomyocytes experienced acute ischemia and hypoxia, ALKBH5 inhibited the m6A methylation of MG53, preventing its degradation. This inhibition suppressed apoptosis and oxidative stress in cardiomyocytes, thereby reducing cell death (Li et al., 2024a). Following the restoration of blood supply, there was a high level of oxidative stress in the tissues, resulting in ischemia/reperfusion (I/R) injury (Metra and Teerlink, 2017). ALKBH5 had protective properties on cell proliferation, injury, and apoptosis during myocardial I/R injury (Li et al., 2022). ALKBH5 could also inhibit I/R-induced autophagy and apoptosis through the EGFR/PI3K/AKT/mTOR pathway (Wang et al., 2024). Additionally, ALKBH5 stabilized SIRT1 mRNA, inhibiting oxidative stress and apoptosis in cardiomyocytes induced by I/R (Liu and Liu, 2023). STAT3 downregulation could aggravate I/R injury in aging cardiomyocytes, while ALKBH5 regulated STAT3 expression by mediating long non-coding RNA (lncRNA) H19/miR-124-3p, thereby alleviating cardiomyocytes damage (Zhang et al., 2023). Therefore, ALKBH5 exerted a protective effect on cardiomyocytes by controlling the stability of various RNAs, potentially reducing the occurrence of cardiac fibrosis following myocardial infarction (MI). However, different results had also been reported. In diabetic cardiomyopathy, ALKBH5 regulated FOXO3 through m6A demethylation in m6A-YTHDF2-dependent manner, activating the CDR1/Hippo signaling pathway ultimately aggravating high glucose-induced cardiomyocytes apoptosis (Shao et al., 2022). ALKBH5 could exacerbate doxorubicin-induced cardiomyocytes apoptosis by regulating AT-Rich Interaction Domain 2 (ARID2) expression (Chen et al., 2024).

ALKBH5 also played a significant role in hepatocytes. In the mouse model of non-alcoholic fatty liver disease (NAFLD), inhibiting the activity of ALKBH5 significantly induced hepatocytes autophagy by reducing the stability of AXL mRNA (Meng et al., 2023).

### 3.2 The impact of ALKBH5 on immune cells

ALKBH5 played crucial roles in regulating the functions of inflammatory cells such as neutrophils and T lymphocytes, which were important in fibrosis. ALKBH5 played important roles in emergency granulopoiesis and neutrophil mobilization. In a mouse model of polymicrobial sepsis induced by cecal ligation puncture (CLP), ALKBH5 promoted G-CSF factor-driven emergency granulopoiesis and neutrophil mobilization by upregulating G-CSFR expression. ALKBH5 depletion significantly impaired the production of immature neutrophils and leaded to higher retention of mature neutrophils in the bone marrow (Liu et al., 2024). Furthermore, in a mouse model of polymicrobial sepsis induced by CLP, ALKBH5 regulated the expression of molecules involved in neutrophil migration, increasing the expression of CXCR2 and NLRP12, which promoted neutrophil migration, while decreasing the expression of PTGER4, TNC, and WNK1, which inhibited neutrophil migration. Deficiency of ALKBH5 impaired neutrophil migration (Liu et al., 2022). It seemed that ALKBH5 could promote the infiltration of neutrophils.

Different types of T lymphocytes also played important roles during the inflammatory phase of fibrosis. CD4^+^ T lymphocytes could produce the cytokine IFN-γ, which further activated macrophages (Mosser and Edwards, 2008). During neuroinflammation, ALKBH5 decreased m6A modification of IFNG mRNA of CD4^+^ T cells, leading to increased mRNA stability, thus promoting the secretion of IFN-γ (Zhou et al., 2021). At the same time, ALKBH5 mediated the stability of CXCL2 mRNA in CD4^+^ T cells through m6A, affecting CXCL2 expression in CD4^+^ T cells, resulting in promoted CD4^+^ T cell response and neutrophil recruitment (Zhou et al., 2021). ALKBH5 had additional functions in regulating the development and function of γδ T cells and mature T cells. ALKBH5 regulated the expression levels of Jagged1 and Notch2 in lymphocytes, changing the Jagged1/Notch2 signaling pathway, affecting the development of γδ T cell precursors, and thus reduce the number of mature γδ T cells (Ding et al., 2022). Furthermore, in a case-control study of lupus erythematosus, it was found that overexpression of ALKBH5 promoted apoptosis of mature T cells and inhibit T cell proliferation (Deng et al., 2022). The interaction between programmed death-ligand 1 (PD-L1) and programmed cell death protein 1 (PD-1) triggered immune inhibitory signals, suppressing the activity of T cells and other immune cells (Perry et al., 2022). ALKBH5 decreased the expression of PD-L1 in glioblastoma (GB) by regulating the stability of ZDHHC3 mRNA. As a result, there was increased infiltration of cytotoxic lymphocytes and pro-inflammatory cytokines in the cerebrospinal fluid (Tang et al., 2022). In a male mouse model of kidney injury, it was found that inhibiting ALKBH5 enhanced the m6A modification and stability of CCL28 mRNA, thereby regulating the recruitment of Treg cells, ultimately alleviating macrophage and neutrophil infiltration (Chen et al., 2023a). In summary, ALKBH5 could regulate the function of neutrophils and various types of T cells, including CD4^+^ T cells, γδ T cells and mature T cells. However, further research was needed to fully understand the impact of ALKBH5-mediated regulation of these immune cell functions on fibrosis.

### 3.3 The impact of ALKBH5 on macrophages

The macrophages derived from monocytes were crucial in fibrosis (Gordon and Taylor, 2005; Misharin et al., 2017). ALKBH5 regulated the function of macrophages through various mechanisms. Firstly, ALKBH5 promoted the recruitment of macrophages (Wei et al., 2022). In glioblastoma multiforme (GBM), ALKBH5 regulated the m6A modification of lncRNA NEAT1, leading to increased expression of interleukin-8 (IL-8), which promoted macrophage recruitment (Dong et al., 2021). Research had also found that ALKBH5 regulated the expression of MAP3K8 in hepatocellular carcinoma (HCC) cells and modulated IL-8 expression through the JNK and ERK pathways, promoting macrophage recruitment (You et al., 2022).

Secondly, ALKBH5 affected macrophage polarization. Activated macrophages could polarize into M1 (proinflammatory) or M2 (anti-inflammatory/pro-fibrotic) macrophages and performed different functions in different conditions and stages (Adhyatmika et al., 2015; Wang Y. et al., 2023). Several studies had shown that ALKBH5 played significant roles in macrophage polarization. A20 was considered as an anti-inflammatory molecule that inhibited inflammation and blocked the activation of various signaling pathways. In the diabetic retinopathy, ALKBH5-mediated m6A modification leaded to decreased A20 expression, ultimately enhancing M1 polarization of retinal microglia (Chen et al., 2022). However, the impact of ALKBH5 on macrophage polarization seemed to be bidirectional (Jiang et al., 2020). Research had found that ALKBH5 promoted monocyte infiltration and M2 polarization through the regulation of CCL5 production in radiation-induced-Hepatic stellate cells (HSCs), thereby promoted radiation-induced liver fibrosis (RILF) (Chen Y. et al., 2023). ALKBH5 could also affect macrophage recruitment and M2 differentiation by regulating the secretion of vascular endothelial growth factor (VEGF) (Zhao et al., 2022). In lung adenocarcinoma (LUAD), ALKBH5 regulated macrophages M2 polarization through CDCA4 (Tan et al., 2024). Therefore, the impact of ALKBH5 on macrophage polarization stilled remain controversial and might be influenced by different conditions and stages.

Besides, ALKBH5 affected MMT and macrophage autophagy, thereby regulating fibrosis. MMT was an important source of myofibroblasts. Under angiotensin II (AngII)-induced hypertension, cardiac macrophages derived from circulating monocytes preferentially underwent MMT. ALKBH5 mediated m6A demethylation of IL-11 mRNA, leading to increased stability and protein level of IL-11, promoting AngII-induced MMT, and thus resulted in cardiac fibrosis and dysfunction (Zhuang et al., 2024). There was also research suggesting that ALKBH5 reduced Slamf7 mRNA stability in an m6A-dependent manner, promoting macrophage autophagy and reducing the secretion of pro-inflammatory cytokines, thereby mediated silica particles-induced pulmonary inflammation (Yin et al., 2024).

### 3.4 The impact of ALKBH5 on endothelial cells

Vascular ECs and angiogenesis played important roles in fibrosis (Fan et al., 2023). Studies suggested that ALKBH5 might regulate the function of vascular ECs and thus modulated angiogenesis. VEGF, WNT signaling pathway, and hypoxia signaling were known regulators of angiogenesis (Carmeliet and Jain, 2011; Cui et al., 2018). Research had shown that overexpression of ALKBH5 increased VEGF-A secretion, promoted EC proliferation, migration, and tube formation, and regulated vessel formation. ALKBH5 knockdown impaired VEGF-A secretion in both *in vitro* and *in vivo* settings in GBM cells, decreasing the pro-angiogenesis ability of GBM cells (Tao et al., 2022; Fan et al., 2024). ALKBH5 overexpression elevated VEGF-A secretion in retinal pigment epitheliums (RPEs), thereby accelerating choroidal neovascularization progression in age-related macular degeneration (Sun et al., 2023). During ischemia, ALKBH5 decreased the m6A level on sphingosine kinase 1 (SPHK1) mRNA in ECs, increasing its stability and protein level, promoting vascular generation after acute ischemic stress (Kumari et al., 2021). ALKBH5 had also been found to regulate other extracellular mediators that affected angiogenesis. For example, connective tissue growth factor (CTGF), a vascular growth factor, showed significantly reduced expression in ALKBH5 knocked-out breast cancer cells (Panneerdoss et al., 2018). Increasing ALKBH5 level promoted angiogenesis by regulating CTGF production. ALKBH5 also influenced angiogenesis by modulating m6A level on non-coding RNA. lncRNA PVT1 promoted vascular generation in lung cancer tissue, and ALKBH5 reduced m6A modification on PVT1, thereby facilitating vascular generation in lung cancer (Shen et al., 2022). ALKBH5 could also affect angiogenesis by regulating circular RNA. In HCC, ALKBH5 regulated m6A modification on circ-CCT3, affecting angiogenesis (Liu et al., 2023). These studies suggested a potential role of ALKBH5 in promoting angiogenesis. However, there were also reports with opposing findings. For instance, it had been found that downregulation of ALKBH5 in vascular ECs under hypoxic conditions regulated the expression of WNT5A in an m6A-dependent manner, increasing its stability and promoting vascular generation phenotypes (Zhao et al., 2021).

### 3.5 The impact of ALKBH5 on fibroblasts

Activation of fibroblasts and their transformation into myofibroblasts were crucial steps in fibrosis (Frangogiannis, 2021). ALKBH5 had been found to regulate the activation of fibroblasts. During the post- MI healing process, ALKBH5 promoted the transformation of cardiac fibroblasts into myofibroblasts and improved collagen repair after MI by enhancing the stability of ErbB4 mRNA (Yang et al., 2023). In a mouse model of silica-induced pulmonary fibrosis, ALKBH5 promoted lung fibroblast activation and silica-induced pulmonary fibrosis via the miR-320a-3p/FOXM1 axis or targeting FOXM1 directly (Sun et al., 2022b).

ALKBH5 had also been found to regulate the transformation of multiple mesenchymal cell types into myofibroblasts. HSCs could transit from a quiescent state to a proliferative myofibroblast phenotype in response to liver injury (Trivedi et al., 2021). *In vitro* and *in vivo* models, including HSCs and clinical cases or CCl4-induced mice liver fibrosis, ALKBH5 triggered PTCH1 activation in an m6A-dependent manner, leading to hedgehog signaling inactivation, which inhibited the transformation of HSCs into myofibroblasts and ameliorated liver fibrosis (Yang et al., 2022). Furthermore, ALKBH5 reduced Drp1 methylation in an m6A-YTHDF1-dependent manner, inhibiting HSC proliferation, and migration, thereby ameliorating liver fibrosis (Wang J. et al., 2023).

Myofibroblasts could also undergo transformation from epithelial cells and macrophages. In a mouse model of renal fibrosis induced by unilateral ureteral obstruction (UUO). Knockdown of ALKBH5 enhanced the expression of mesenchymal markers, such as α-smooth muscle actin and snail, while overexpression of ALKBH5 increased the expression of the epithelial adhesion molecule E-cadherin and decreases snail expression, alleviating renal fibrosis (Ning et al., 2020), suggesting that ALKBH5 might play an important role in the EMT process. MMT was another important source of myofibroblasts. Under AngII-induced hypertension, ALKBH5 mediated the m6A demethylation of IL-11 mRNA, leading to increased stability and protein level of IL-11, promoting AngII-induced MMT, resulting in cardiac fibrosis and functional impairment (Zhuang et al., 2024).

## 4 The impact of ALKBH5 on organ fibrosis

### 4.1 The impact of ALKBH5 on pulmonary fibrosis

The occurrence of pulmonary fibrosis could be caused by factors such as radiation, inhalation of substances (crystalline silica, PM2.5), and chronic inflammation. ALKBH5 had been found to regulate pulmonary fibrosis in various animal models. Long-term exposure to crystalline silica could cause chronic respiratory disease and lead to silicosis, a disease characterized by diffuse fibrosis of the lung tissue (Hornung et al., 2008). Studie had discovered that ALKBH5 could regulate fibroblast-to-myofibroblast differentiation via the miR-320a-3p/FOXM1 axis or targeting FOXM1 directly, promoting silica-induced pulmonary fibrosis (Sun et al., 2022b). These studies suggested that ALKBH5 might promote the occurrence of pulmonary fibrosis.

However, some studies had also found that downregulating ALKBH5 might promote the development of pulmonary fibrosis. Exposure to 1-nitropyrene (1-NP) could cause pulmonary fibrosis in mice. Research had found that 1-NP promoted ALKBH5 degradation, regulating lung epithelial cells senescence through FBXW7 m6A modification, ultimately leading to 1-NP-induced pulmonary fibrosis (Li SR. et al., 2024). Radiation-induced pulmonary fibrosis (RIPF) was a common complication of thoracic radiation therapy (Hanania et al., 2019). ALKBH5 mediated the demethylation of IL-6 mRNA by silencing zinc finger and BTB domain-containing protein 7B (Zbtb7b), inhibiting its nuclear export, and suppressing the production of IL-6 in the lung, thereby slowing the development of RIPF (Zhao et al., 2020). In silica-induced pulmonary fibrotic tissue, ALKBH5 reduced Slamf7 through an m6A-dependent mechanism, promoting macrophage autophagy and reducing the secretion of pro-inflammatory cytokines, thereby inhibiting silica-induced lung inflammation (Yin et al., 2024). Similar to inhalation of crystalline silica, long-term exposure to PM2.5 could lead to pulmonary fibrosis. Knockdown of ALKBH5 significantly upregulated the YAP1 signaling pathway in NIH3T3 cells and lung tissue, promoting extracellular matrix deposition in PM2.5 exposure-induced pulmonary fibrosis of mice (Zhang et al., 2022). Therefore, the impact of ALKBH5 on pulmonary fibrosis still requires further elucidation (Table 2).

**TABLE 2:** The impact of ALKBH5 on organ fibrosis.

Organ	Cells	Expression^b^	Target RNAs	Target pathways	Functions	Ref
Lung	Fibroblasts	Up	FOXM1	Induce fibroblast-to-myofibroblast differentiation	Promote silica-induced pulmonary fibrosis	Sun et al. (2022b)
	—	Up	Zbtb7b	Alleviate radiation pneumonia	Inhibit RI pulmonary fibrosis	Zhao et al. (2020)
	Macrophages	Up	Slamf7	Promoting macrophages autophagy and reducing cytokines secretion	Inhibit silica-induced pulmonary fibrosis	Yin et al. (2024)
	Lung tissue	Down	miRNA	YAP1/TAZ/P4HA2	Inhibit PM2.5-induced pulmonary fibrosis	Jin et al. (2020)
Liver	Hepatocytes	Down	AXL	ERK/LKB1/AMPK	Slow the NAFLD-fibrosis process	Meng et al. (2023)
	HSCs	Up	Drp1	Inhibit HSC proliferation and migration	Ameliorate fibrosis	Wang et al. (2023b)
			PTCH1	Hedgehog	Ameliorate liver fibrosis	Yang et al. (2022)
	RI-HSCs	Up	TIRAP	NF-κB and JNK/Smad2	Enhance RI liver fibrosis	Chen et al. (2023b)
Heart	Fibroblasts	Up	ErbB4	Induce fibroblast activation	Promote post-MI fibrotic repair	Yang et al. (2023)
	ECs	Up	SPHK1	SPHK/eNOS-AKT	Improve fibrosis caused by MI	Kumari et al. (2021)
	Cardiomyocytes	Up	YTHDF1	ALKBH5-m6A-YTHDF1-YAP	Ameliorates fibrosis caused by MI	Han et al. (2021)
		Up	FOXO3	CDR1as/Hippo	Promote glucose-induced cardiac fibrosis	Shao et al. (2022)
		Down	ARID2	Enhance cardiomyocytes apoptosis	Promote doxorubicin-induced cardiac fibrosis	Chen et al. (2024)
Kidney	Tubular epithelial cells	Up	—	Affect the EMT process	Alleviate UUO-induced renal fibrosis	Ning et al. (2020)
	Glomerular mesangial cells	Down	TRIM13	Inhibit inflammation and excessive proliferation	Slow the development of chronic glomerulonephritis	Hu et al. (2024)
	Tubular epithelial cells	Down	CCL28	CCL28/Treg/inflammatory cell axis	Alleviate I/R-induced acute kidney injury and renal fibrosis	Chen et al. (2023a)

NAFLD, non-alcoholic fatty liver disease; HSCs, hepatic stellate cells; RI, radiation-induced; MI, myocardial infarction; ECs, endothelial cells; EMT, Epithelial-Mesenchymal Transition; UUO, unilateral ureteral obstruction; I/R, ischemia/reperfusion.

^a^ ALKBH5 expression.

### 4.2 The impact of ALKBH5 on liver fibrosis

The occurrence of liver fibrosis could be caused by multiple factors, and ALKBH5 played significant roles in this process (Paternostro and Trauner, 2022; Chen et al., 2023c). In a mouse model of NAFLD, inhibiting ALKBH5 regulated hepatic autophagy flux, thereby alleviating hepatic steatosis and fibrosis (Meng et al., 2023). HSCs were the major fibrotic cells in the liver and could transform into activated myofibroblast-like cells upon activation, secreting extracellular matrix components involved in the formation of liver fibrosis and structural remodeling (Tsuchida and Friedman, 2017). Mitochondrial homeostasis played an important role in the progression of liver fibrosis (An et al., 2020; Wu et al., 2023). ALKBH5 inhibited mitochondrial fission and HSC proliferation and migration by reducing Drp1 methylation in an m6A-YTHDF1-dependent manner, ultimately ameliorating liver fibrosis (Wang J. et al., 2023). Furthermore, ALKBH5 could mediate the activation of PTCH1 through an m6A-dependent mechanism. Upregulation of PTCH1 leaded to inactivation of the Hedgehog signaling pathway, inhibiting HSC activation and ameliorating liver fibrosis (Yang et al., 2022). It seemed that ALKBH5 ameliorated liver fibrosis. However, in radiation-induced HSCs (radiation could induce HSC activation and promote RILF, which was a common complication of HCC radiotherapy (Kim and Jung, 2017). ALKBH5 mediated the demethylation of toll-interleukin 1 receptor domain-containing adaptor protein (TIRAP) mRNA through m6A modification, activating the downstream NF-κB and JNK/Smad2 pathways, and promoting radiation-induced HSC activation. Knockdown of ALKBH5 significantly alleviated RILF in mice (Chen Y. et al., 2023). These suggested that ALKBH5 could improve RILF (Table 2).

### 4.3 The impact of ALKBH5 on cardiac fibrosis

The injured myocardium was replaced by scar tissue, and excessive myocardial injury could lead to cardiac fibrosis (Bergmann et al., 2009). Myocardial ischemia and hypoxia were the primary causes of myocardial injury and cardiac fibrosis. During acute ischemia and hypoxia in the myocardium, ALKBH5 could influence the extent of MI and cardiac fibrosis through multiple mechanisms (Wang et al., 2021; Cheng et al., 2022; Li et al., 2024b). After the ischemic myocardium was reperfused, ALKBH5 inhibited H/R-induced autophagy and apoptosis in cardiomyocytes through pathways such as EGFR/PI3K/AKT/mTOR (Liu and Liu, 2023; Wang et al., 2024). During the repair phase of MI, ALKBH5 enhanced the stability of ErbB4 mRNA through m6A demethylation, promoting the transformation of fibroblasts into myofibroblasts and improving post-MI fibrotic repair (Yang et al., 2023). ALKBH5 also contributed to maintaining post-acute ischemic stress-induced angiogenesis by reducing SPHK1 mRNA m6A methylation, thereby improving post-MI fibrosis progression (Kumari et al., 2021). While it had long been believed that mammalian cardiomyocytes had limited ability to re-enter the cell cycle and primarily relied on fibrotic repair after myocardial injury (Bergmann et al., 2009; Weinberger and Riley, 2024), the concept of cardiac regeneration had gained attention among researchers with advancements in scientific technology (Weinberger and Riley, 2024). Studies had reported that ALKBH5, through the ALKBH5-m6A-YTHDF1-YAP axis, regulated cardiomyocytes re-entry into the cell cycle and promoted cardiomyocyte proliferation after MI (Han et al., 2021). These studies suggested that ALKBH5 was beneficial for the repair of ischemia-induced myocardial injury and improvement of cardiac fibrosis.

Diabetic cardiomyopathy was also a common cause of cardiac fibrosis (Zhang et al., 2019). ALKBH5 activated FOXO3 through m6A demethylation in an m6A-YTHDF2-dependent manner, activating the CDR1/Hippo signaling pathway, ultimately aggravating high glucose-induced cardiomyocytes apoptosis (Shao et al., 2022). Drug toxicity could also lead to cardiac fibrosis, with drugs like doxorubicin exhibiting dose-dependent cardiac toxicity that could result in heart failure and interstitial fibrosis (Deng et al., 2007). ARID2, serving as a downstream effector of ALKBH5 in cardiomyocytes, regulated the DNA damage response and enhanced doxorubicin-induced cardiomyocyte apoptosis, ALKBH5 could affect the role of doxorubicin-induced cardiac dysfunction, remodeling, and cardiomyocyte apoptosis by regulating ARID2 expression (Chen et al., 2024). Under AngII-induced hypertension, ALKBH5 mediated m6A demethylation of IL-11 mRNA to increase the stability IL-11, promoting AngII-induced MMT, and resulted in cardiac fibrosis (Zhuang et al., 2024).

### 4.4 The impact of ALKBH5 on renal fibrosis

In a mouse model of renal fibrosis induced by UUO, overexpression of ALKBH5 could increase the expression of the renal epithelial adhesion molecule E-cadherin while decreasing the expression of snail, thereby alleviating UUO-induced renal fibrosis by affecting the EMT process. This suggested that ALKBH5 could slow down the progression of kidney fibrosis (Ning et al., 2020). However, different results had also been reported. For example, studies found that inhibiting ALKBH5 reduced glomerular inflammation and excessive proliferation of mesangial cells through the modification of TRIM13-m6A in glomerular mesangial cells, thereby slowing the development of chronic glomerulonephritis. This suggested that inhibiting ALKBH5 could slow down the progression of kidney fibrosis (Hu et al., 2024). In the I/R-induced renal injury model of male mice, researchers found that knocking down ALKBH5 increased the expression of CCL28 through m6A modification, regulated the downstream CCL28/Treg/inflammatory cell axis, thereby alleviating I/R-induced acute kidney injury and renal fibrosis. This also suggested that inhibiting ALKBH5 could slow down the progression of renal fibrosis (Chen et al., 2023a). Therefore, the effect of ALKBH5 on renal fibrosis needed further study (Table 2).

## 5 Applications of ALKBH5 research: inhibitors

Several inhibitors of ALKBH5 had been discovered in current research. 2OG-dependent oxygenase’s inhibitors such as NOG, PDCA, and HIF PHD inhibitor IOX3 could weakly inhibit ALKBH5 activity by competing with 2OG, but they lack selectivity (Aik et al., 2014). Among them, 4-PDCA exhibited the strongest inhibitory effect, while NOG had the weakest inhibitory effect. The imidazobenzoxazin-5-thione (MV1035), a novel sodium channel blocker, partially overlapped with the 2OG binding site of ALKBH5. MV1035 inhibited the catalytic activity of ALKBH5 by competing with 2OG at the active site, reducing GBM cells migration and invasiveness (Malacrida et al., 2020; Malacrida et al., 2022). Dexmedetomidine had been found to inhibit ALKBH5 expression *in vitro* and decrease inflammation cytokine production of LPS-treated HK-2 cells (Zhu and Lu, 2020). However, these inhibitors were not primarily targeted at ALKBH5 demethylase.

Specific inhibitors targeting ALKBH5 were gradually being discovered. Researchers identified two novel ALKBH5 inhibitors, Ena15 and Ena21. Ena21 was a selective competitive inhibitor of ALKBH5, which could compete with 2OG for the active site of ALKBH5 and inhibit ALKBH5 activity, while Ena15 was a non-competitive inhibitor of ALKBH5. Both of them could inhibit the growth activity of GBM (Takahashi et al., 2022). Pyrazolo [1,5-a]pyrimidine Derivative DDO-2728 had also been found to act as a selective inhibitor of ALKBH5, significantly suppressing tumor growth in the MV4-11 xenograft mouse model (Wang YZ. et al., 2023). A drug with a 1-phenyl-1H-pyrazole scaffold called 20m, which had been identified as an effective, selective, and cell-active ALKBH5 inhibitor (Fang et al., 2022). Scientists had also shared a novel small molecule RNA demethylase ALKBH5 inhibitor for targeted cancer therapy (Sabnis, 2021). The selection of ALKBH5 inhibitors specific to specific cells or even specific mechanisms of action would be the focus of future research, relying on a deeper understanding of the mechanisms of ALKBH5 action.

In fact, there was a natural inhibitor of ALKBH5 in cellular environments: citrate. Citrate molecules bound to ALKBH5 demethylase, displacing the metal ions and 2OG in ALKBH5, thereby inhibiting its demethylation activity (Xu et al., 2014). Chlorogenic acid (CGA), a naturally occurring plant component, could inhibit ALKBH5 activity to regulate autophagy and alleviate hepatic steatosis (Meng et al., 2023) (Table 3).

**TABLE 3:** Applications of ALKBH5 research: inhibitors.

Medicine	Mechanisms	Selectivity	Diseases	Functions	Ref
MV1035	Competitive inhibition	Yes	GBM	Reduce GBM cells migration and invasiveness	Malacrida et al. (2020), Malacrida et al. (2022)
Dexmedetomidine	Repression	No	Sepsis	Suppress inflammation	Zhu and Lu (2020)
Ena15	Non-competitive inhibition	Yes	GBM	Inhibit the growth activity of GBM	Takahashi et al. (2022)
Ena21	Competitive inhibition	No	GBM	Inhibit the growth activity of GBM	Takahashi et al. (2022)
DDO -2728	Competitive inhibition	Yes	AML	Suppress tumor growth	Wang et al. (2023c)
CGA	Repression	No	NAFLD	Alleviate hepatic steatosis	Meng et al. (2023)

MV1035, imidazobenzoxazin-5-thione; GBM, glioblastoma multiforme; AML, acute myeloid leukemia; CGA, chlorogenic acid; NAFLD, non-alcoholic fatty liver disease.

## 6 Conclusion

ALKBH5 can regulate the functions of various cells, including parenchymal cells (hepatocytes and cardiomyocytes), immune cells (neutrophils and T cells), macrophages, endothelial cells, and fibroblasts, through different signaling pathways and mechanisms, ultimately affecting tissue fibrosis in organs such as the heart, liver, lung and kidney. The impact of ALKBH5 on fibrotic outcomes varies and may be influenced by different cell types, stimulation conditions, and research models. The focus of future research will be how to select ALKBH5 inhibitors targeting specific cells or even specific mechanisms of action, which relies on a deeper understanding of the mechanisms of ALKBH5.
